# Dynamic behaviour of platinum and copper dopants in gold nanoclusters supported on ceria catalysts

**DOI:** 10.1038/s42004-023-01068-0

**Published:** 2023-12-18

**Authors:** Nicole Müller, Rareş Banu, Adea Loxha, Florian Schrenk, Lorenz Lindenthal, Christoph Rameshan, Ernst Pittenauer, Jordi Llorca, Janis Timoshenko, Carlo Marini, Noelia Barrabés

**Affiliations:** 1 Institute of Materials Chemistry, TU Wien, Getreidemarkt 9/165, 1060 Vienna, Austria; 2 Chair of Physical Chemistry, Montanuniversität Leoben, Franz-Josef-Straße 18, 8700 Leoben, Austria; 3 Institute of Analytics, TU Wien, Getreidemarkt 9/165, 1060 Vienna, Austria; 4 Institute of Energy Technologies and Department of Chemical Engineering, Universitat Politècnica de Catalunya, EEBE, Eduard Maristany 10-14, 08019 Barcelona, Spain; 5 Department of Interface Science, Fritz-Haber Institute of the Max Planck Society, 14195 Berlin, Germany; 6 ALBA Synchrotron Light Facility, Carrer de la Llum 2-26, 08290 Cerdanyola del Valles, Barcelona Spain

**Keywords:** Nanoparticles, Heterogeneous catalysis

## Abstract

Understanding the behaviour of active catalyst sites at the atomic level is crucial for optimizing catalytic performance. Here, the evolution of Pt and Cu dopants in Au_25_ clusters on CeO_2_ supports is investigated in the water-gas shift (WGS) reaction, using operando XAFS and DRIFTS. Different behaviour is observed for the Cu and Pt dopants during the pretreatment and reaction. The Cu migrates and builds clusters on the support, whereas the Pt creates single-atom active sites on the surface of the cluster, leading to better performance. Doping with both metals induces strong interactions and pretreatment and reaction conditions lead to the growth of the Au clusters, thereby affecting their catalytic behaviour. This highlights importance of understanding the behaviour of atoms at different stages of catalyst evolution. These insights into the atomic dynamics at the different stages are crucial for the precise optimisation of catalysts, which ultimately enables improved catalytic performance.

## Abstract (web summary)

Understanding the atomic dynamics of active catalyst sites is crucial for the precise optimization of catalyst performance. Here, the authors employ operando XAFS and DRIFTS to study the dynamics of the mobility of platinum and copper dopants in bimetallic and trimetallic gold nanoclusters supported on ceria, using the water-gas shift process as a model reaction.

## Introduction

The desire for greener energy has increased significantly in recent years, encouraging research towards renewable fuel sources. Hydrogen has emerged as a promising fuel source due to its high energy content. One way to create clean energy hydrogen is the water-gas shift (WGS) reaction, where carbon monoxide (CO) and water (H_2_O) are transformed into hydrogen (H_2_) and carbon dioxide (CO_2_)^1,2^. Understanding the basic structure of a catalyst at the atomic level is essential for making and improving WGS catalysts. It has been previously reported that the particle size and structure, as well as the metal-oxide interface, is essential for achieving a high activity and selectivity already at lower temperatures^3,4^. An emerging class of functional nanomaterials with atomic precision, well-defined molecular structure, and intriguing molecular-like properties are ligand-protected metal nanoclusters. The outstanding size control during the cluster synthesis, opens more opportunities for accurate studies of size-dependent properties, atomic structure effects, and reaction mechanisms in catalysis^5,6^. There are a large number of studies exploring the use of copper (Cu), gold (Au), platinum (Pt), palladium (Pd), and nickel (Ni) nanoparticles as active catalysts for the WGS reaction^7–12^. Furthermore, between different oxides investigated as support, CeO_2_ appeared as optimal support for the WGS reaction as well as for the stabilization of metal nanoparticles^13^.

Copper-based catalysts supported on CeO_2_ have been extensively studied for the WGS reaction, but there are still some drawbacks regarding stability, selectivity, and reaction kinetics. As a result, gold and platinum-based catalysts supported on various materials such as TiO_2_, ZrO_2_, and ceria (CeO_2_)^14–17^ have emerged as active WGS catalysts that can compete with conventional Cu/ZnO/Al_2_O_3_ catalysts^18–20^. However, Au catalysts also suffer from stability and selectivity problems in some cases. Therefore, alloyed nanoparticles that combine Au, Cu, and Ag especially, as well as Pt and Pd, have been investigated as active sites, overcoming the stability and reactivity problems due to the synergistic effects between the metals^21^.

Despite the acceptance of the general mechanism for monometallic catalysts, the nature of the catalytically active metal species still needs to be fully understood. When using ceria as a support, CO is adsorbed on the metal nanoparticles, while H_2_O is activated on the oxygen vacancies on the surface of ceria, and subsequent steps take place at the metal–CeO_2_ interface^14^. The activity in Au and Pt supported on CeO_2_ has been associated with non-metallic (cationic, Au^δ+^) species that are strongly associated with the surface CeO_2_ oxygen vacancies^22,23^. However, several contradictions were observed in the works of different groups^24^.

Reina et al. found that both Pt and Au remained in their unoxidized form during WGS. For Pt catalysts, a reconstruction of the nanoparticles to cuboctahedral particles was reported, but their size did not change significantly. Au, on the other hand, showed more dynamic development, undergoing size/structure modifications such as particle-splitting or agglomeration. Nevertheless, both metals remained in their unoxidized state, even when not in a metallic environment^25^. Frenkel’s group reports on the dynamics of the Pt atoms at the perimeter of the nanostructures, further solidifying the argument that the dynamic sites are essential for catalytic activity in the case of Pt^26^. Behm’s group found that the oxidation state of Au atoms depends heavily on pretreatment, but the active species is the metallic gold arranged in nanometre sized particles^27^. Therefore, it is crucial to control and understand the active sites at an atomic level to ensure the success of the process.

Gold nanoclusters have been shown to exhibit unique reactivity and stability when heteroatom doping is applied. Bimetallic active sites with atomic precision can be created by selectively doping the clusters. The positioning of dopant atoms within the cluster structure has been identified to depend on the nature of the dopant. For instance, in the case of the Au_25_(SR)_18_ nanocluster, which consists of a core of 13 Au atoms stabilized by staple units (-S-Au-S-Au-S-) around the core, the dopant atoms can be located at the core or the staples^28–30^. Previous works using crystallographic and spectroscopic techniques, correlated with theoretical calculations, revealed that specifically Pt and Pd occupy the centre, Ag resides in the outer core shell, and Cu is present in the protecting Au(I)-thiolate staple motifs that surround the core^28,30,31^. These findings have implications for the tuning of the reactivity and stability of gold nanoclusters through heteroatom doping, which could lead to the development of tailored and efficient catalysts for various applications^28,32^.

Our previous research has shed light on the role of cluster structure and support material in influencing the stability and reactivity of nanoclusters in various gas and liquid phase reactions, as revealed by operando X-ray absorption spectroscopy (XAS) and infrared (IR) measurements. We have also demonstrated the potential for heteroatom doping to modulate the activity and stability of nanoclusters. For example, we have elucidated the dynamic structure of Pd-doped Au_25_ nanoclusters during pre-treatment and CO oxidation reaction, which resulted in the formation of isolated Pd single sites on the Au cluster surface^33^. Similarly, we observed that Ag doping of Ag_x_Au_25-x_(SR)_18_ nanoclusters supported on ITQ2 zeolite enhanced their catalytic performance by promoting the formation of AgAu alloy sites, as detected by X-Ray absorption fine structure spectroscopy (XAFS) under both pre-treatment and reaction conditions^34^. Moreover, by tuning the number of Co doping atoms in Au_25_ nanoclusters supported on CeO_2_, we were able to create different CoAu nanoalloy structures with distinct catalytic performance. Our XAFS and infrared (IR) spectroscopy studies provided insight into the atomic mobility of the Co atoms within the cluster structure under pre-treatment and reaction conditions^35^.

In this study, we investigate the intricate atomic-level structural dynamics and synergies of Cu, Pt, and Au within mono-, bi-, and trimetallic nanoclusters under realistic reaction conditions. These nanoclusters were supported on CeO_2_ and employed as catalysts in the WGS reaction, with their behaviour extensively analysed through operando XAFS and IR spectroscopic measurements.

## Results and discussion

**Fig. 1:**
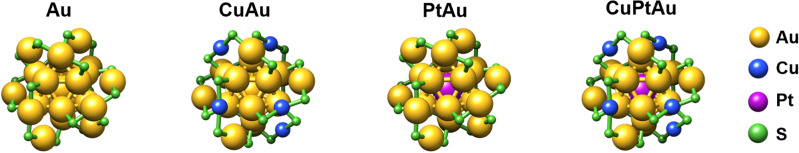
Structure of the nanoclusters. Structure of the (doped) nanoclusters based on reported crystal structures.

We synthesized and characterized four different types of nanoclusters: monometallic Au_25_(SC_2_H_4_Ph)_18_ (named Au), bimetallic PtAu_24_(SC_2_H_4_Ph)_18_ (named PtAu), Cu_x_Au_25-x_(SC_2_H_4_Ph)_18_ (named CuAu), and trimetallic Cu_x_PtAu_24-x_(SC_2_H_4_Ph)_18_ (named PtCuAu). Their synthesis and characterization are described in detail in the Supporting Information (Figs. S1 and S2). For clarification, the structure of the synthesized clusters is represented in Fig. 1, indicating the different locations of the doped atoms depending on the type of metal based on reported structural studies and previous experiences^31,36,37^. In the case of Pt, only one atom is incorporated in the centre of the core structure of the metal, whereas in the case of Cu, several atoms can be doped (1–5), mainly located in the staple units around the core. The results presented assume a normal distribution of the amount of Cu dopant. A similar migration of Pt from the core to the surface of the cluster, as in the case of Pd, is expected due to the similar behaviour of Pt and Pd during doping, creating a single atom active site^28–30^. The catalysts were supported on CeO_2_ (named Ce) by wet impregnation of ceria, yielding different loadings (%wt) depending on the nanocluster (further details are provided in the Supporting Information in Table S1).

### Catalytic activity of bimetallic cluster catalyst

**Fig. 2:**
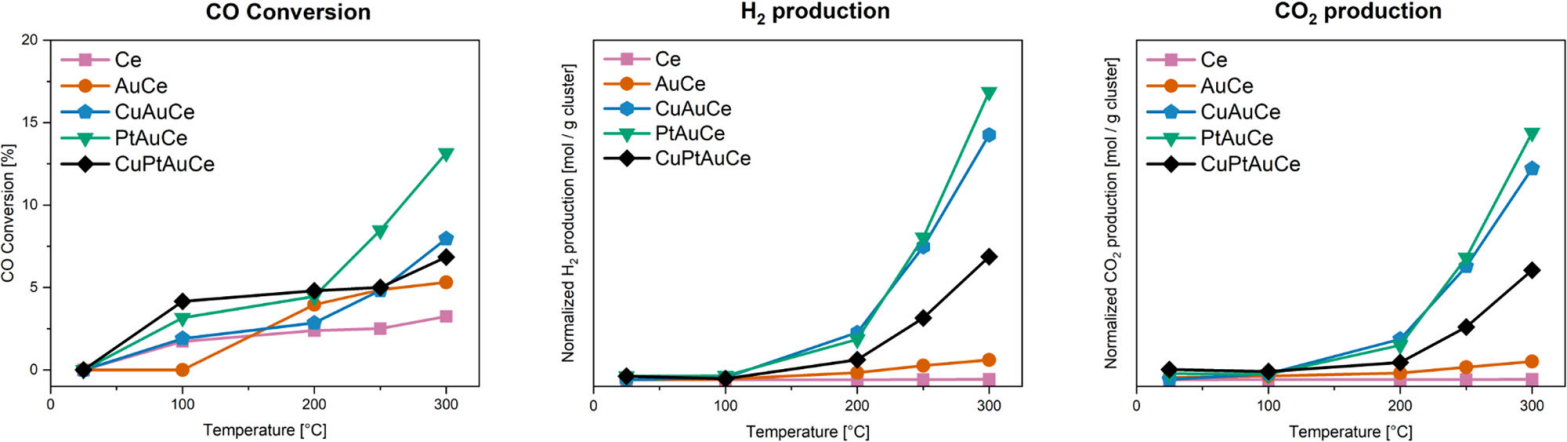
Catalytic activity studies. Catalytic activity of the nanocluster catalysts (pretO_2_ and pretH_2_) in the water-gas shift reaction from RT to 300 °C (reaction conditions in SI).

The effects of doping on the reactivity of nanocluster catalysts were investigated in the WGS reaction. Based on previous experience, a combination of oxidative/reductive pretreatment was performed before the reaction. The catalytic test in the WGS reaction was started at room temperature and then temperature was gradually increased to 300 °C. The results are presented in Fig. 2, which shows the CO conversion and the H_2_ and CO_2_ production at different reaction temperatures. The incorporation of metal nanoclusters increased the catalytic activity compared to the support alone (CeO_2_). Up to a temperature of 200 °C, the catalytic activity showed a consistent pattern for all catalyst types. Both the trimetallic and PtAuCe catalysts showed similar activity values, closely followed by the monometallic catalysts. Surprisingly, the CuAuCe catalyst showed the same activity as the support material. However, with increasing temperature, there was a remarkable shift in the activity trend. At 250 °C, the bimetallic cluster catalyst showed a significant increase in activity compared to their monometallic and trimetallic counterparts. This trend continued as the temperature was further increased to 300 °C. In terms of activity and selectivity, the bimetallic PtAuCe catalysts was found to give the highest CO conversion and yields in the production of H_2_ and CO_2_. It was closely followed by the CuAuCe catalysts, with the trimetallic CuPtAuCe catalysts lagging slightly behind. Surprisingly, the bimetallic nanoclusters outperformed their trimetallic counterparts in terms of yield, CO conversion, and selectivity towards the desired products. These nanoclusters achieved an impressive 50% selectivity for each desired product. In contrast, the trimetallic nanoclusters exhibited lower catalytic activity (similar conversion as CuAuCe, but much lower selectivity). Finally, the monometallic AuCe showed a selectivity only slightly higher than the support alone. It appears that the synergistic effects of Au-Pt, Cu-Au, and Cu-Pt-Au vary depending on the specific combination of metal atoms within the Au cluster structure.

Stability assessments, including multiple catalytic runs (see Fig. S3), also shed light on this phenomenon. The PtAuCe catalysts showed remarkable stability in both activity and selectivity over consecutive runs, closely followed by the CuAuCe catalysts. However, the trimetallic catalysts exhibited a significantly decreasing catalytic performance over time. This observed dynamic behaviour suggests that there are intrinsic changes within the cluster structure that influence their catalytic properties.

### Structure dynamics: HRTEM, XAFS, and CO-IR studies

**Fig. 3:**
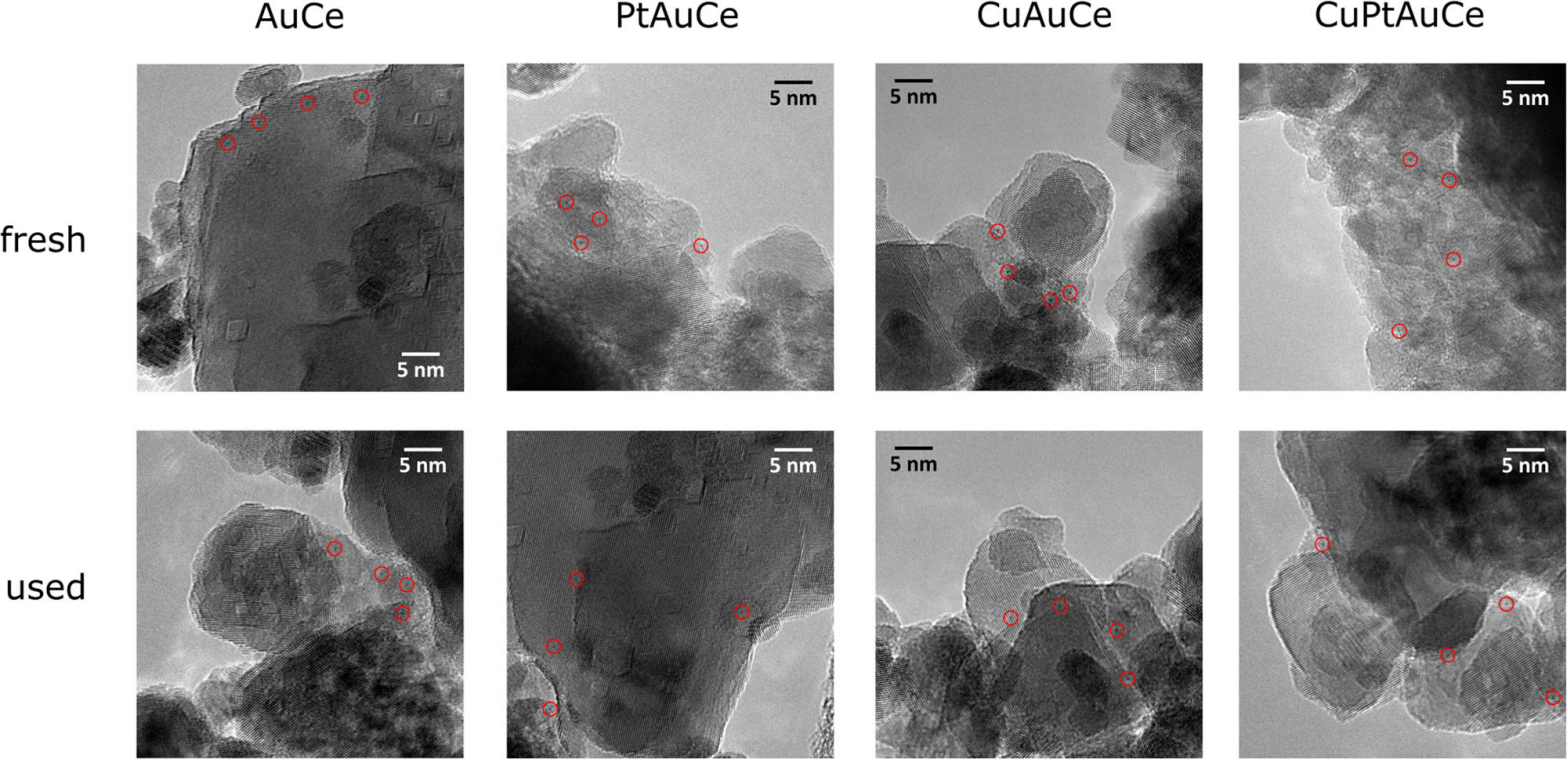
HRTEM images of the catalysts. HRTEM images of the nanocluster catalysts as prepared (fresh) and after the WGS reaction (used).

High-resolution transmission electron microscopy (HRTEM) was carried out on both fresh and reaction samples to assess cluster stability during the reaction. For the monometallic sample, high-angle annular dark-field scanning transmission electron microscopy (HAADF-STEM) was also attempted. Strikingly, there is no visible presence of gold clusters in these HAADF-STEM images. This suggests that the gold particles are indeed small, effectively ruling out the possibility of significant gold agglomeration. However, it is essential to consider that the *Z*-contrast between ceria and gold is relatively low, making it challenging to detect the gold clusters via HAADF-STEM. Thus, we can only confidently affirm the absence of substantial gold agglomeration. For a more in-depth examination of the gold clusters, HRTEM analysis was performed, consistent with our approach for all samples (Fig. 3). In these HRTEM images, alongside well-defined lattice fringes of ceria corresponding to the (111), (200), and (220) crystallographic planes of CeO_2_ at 3.1, 2.8, and 1.9 Å, respectively, we observe darker regions that can be attributed to the presence of gold clusters. Some of these regions are enclosed within red circles in the images for clarity. Due to their small size, energy dispersive X-ray analysis proves ineffective in conclusively confirming the presence of gold. Remarkably, these Au clusters exhibit excellent dispersion over the ceria support and appear to be firmly anchored to the surface. These subnanometric Au clusters defy precise size determination due to the resolution limitations of our instrument, which lacks aberration correction. Importantly, the dimensions of these Au clusters remain unchanged even after the WGS reaction, as shown in Fig. 3.

The sample containing Pt closely resembles the monometallic sample, with the AuPtCe clusters maintaining their subnanometric size both in the fresh sample and after the reaction. Remarkably, there is no evidence of cluster sintering or agglomeration. The same pattern holds true for the sample containing AuCu clusters. In the fresh sample, the AuCu clusters are uniformly distributed throughout the ceria crystallites and retain their subnanometric dimensions after undergoing the WGS reaction. Again, no agglomeration is observed. These observations are consistent across the samples containing Au, Pt, and Cu individually. Whether the fresh samples are examined or their counterparts after the reaction, the characteristics remain virtually identical with subnanometric clusters consistently present. In addition, the clusters show exceptional dispersion on the ceria support and there is no evidence of metal agglomeration in any of the cases. Furthermore, both the size and dispersion of the clusters remain unchanged after the WGS reaction. In summary, our results show that (i) subnanometric clusters are a consistent feature in all samples, regardless of their composition; (ii) the clusters exhibit excellent dispersion on the ceria support, with no evidence of metal agglomeration in any scenario; and (iii) the size and dispersion of the clusters remain unchanged after the WGS reaction.

**Fig. 4:**
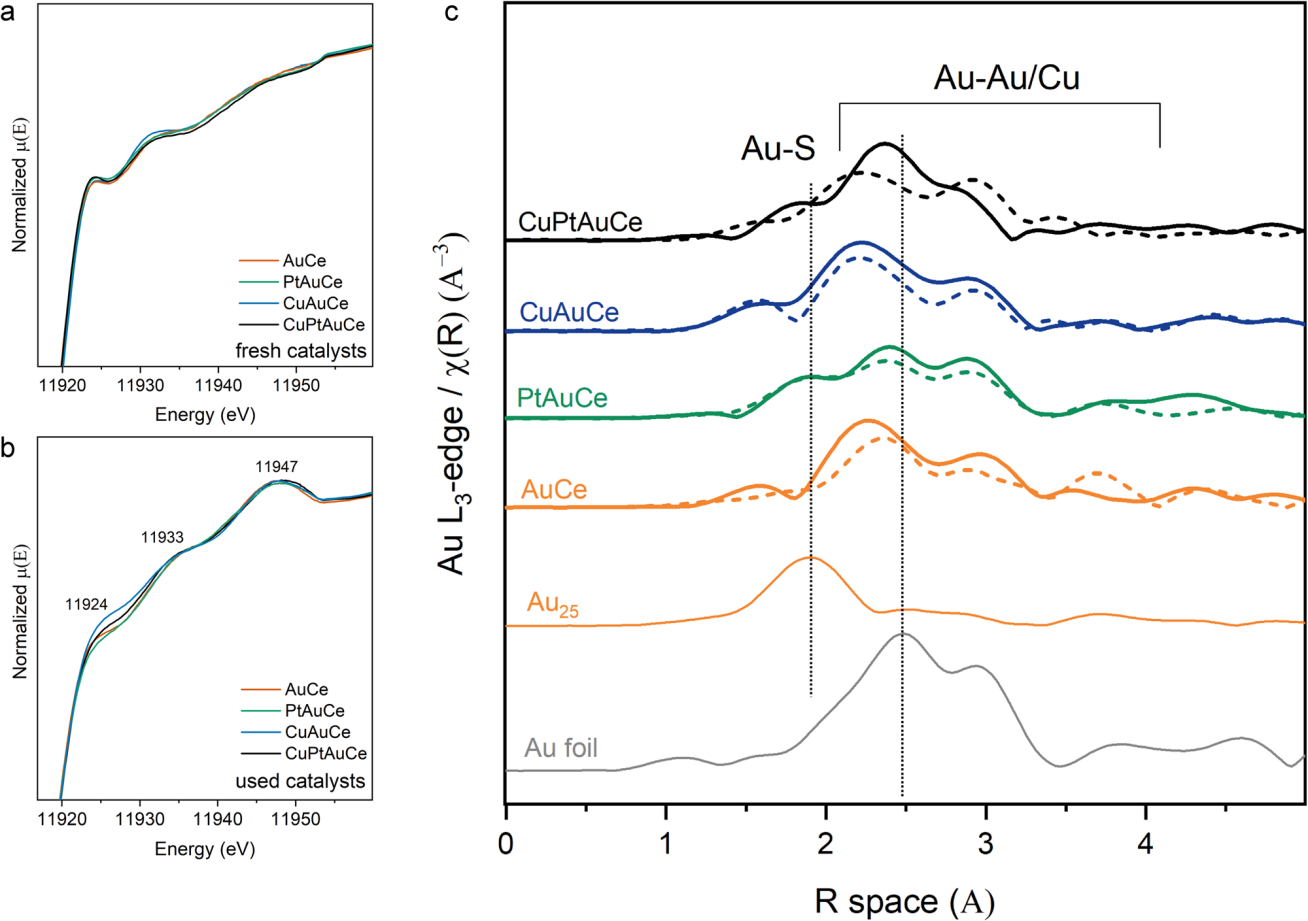
XAFS of the Au L_3_-edge. **a**, **b** XANES spectra at Au L_3_-edge of the nanocluster catalysts **a** before the reaction (without pretreatment) and **b** after WGS reaction (used); **c** EXAFS R-Space of the catalysts after both pretreatments (pretO_2_ and pretH_2_) in dotted lines and after WGS reaction (solid lines) and Au_25_ cluster and Au foil as references.

To gain a comprehensive understanding of the dynamic behaviour of the metal atoms in the clusters, we have performed operando XAFS studies. To evaluate and validate the structural stability of these nanoclusters under different doping conditions, we have first analysed at the Au-L_3_ edge. Figure 4a, b show the X-ray absorption near edge structure spectroscopy (XANES) spectra of the clusters before and after the reactions. Fine variations can be seen in the spectra before the reaction, which are due to changes in both the structure and the electronic properties caused by the introduction of different dopants^38–41^.

However, when we compare the catalysts used (as shown in Fig. 4b), we notice distinctive changes in the intensity of the white lines at 11924 eV. These variations may be linked to modifications in the 5d occupied/unoccupied (d-hole) states and signify a transfer of charge between gold (Au) and other metals. A lower intensity may indicate a reduced number of vacancies in the 5d band due to interactions with platinum (Pt), while the opposite scenario may occur with copper (Cu) interactions. Furthermore, aside from the removal of Au-S bonds during pretreatment, the observed differences may also stem from alterations in the migration or redistribution of doped atoms within the Au_25_ structure.

Figure 4c shows the Au L_3_-edge extended X-Ray absorption fine structure spectroscopy (EXAFS) spectra in R-space, with a detailed fitting presented in Supporting Information Tables S2–S4 and Fig. S5. We examined the catalysts both prior to the reaction (following both pretreatment steps) and after the WGS reaction. During the reaction, an evolution of the cluster structure can be observed, which is characterized by a slight increase in the Au-Au coordination numbers (CN). In particular, for the bimetallic clusters, the CN increases from 6 to 7–8, indicating a detectable shift. However, for the trimetallic clusters, the CN increases to a more remarkable extent, reaching a value of CN = 10, which is a significantly increased coordination number. This distinct behaviour is linked to the presence of different heteroatom doping. To gain a deeper understanding of these dynamics, we conducted Cu K-edge XAFS experiments, allowing us to analyse the evolution of the doping atoms at each step of the process.

**Fig. 5:**
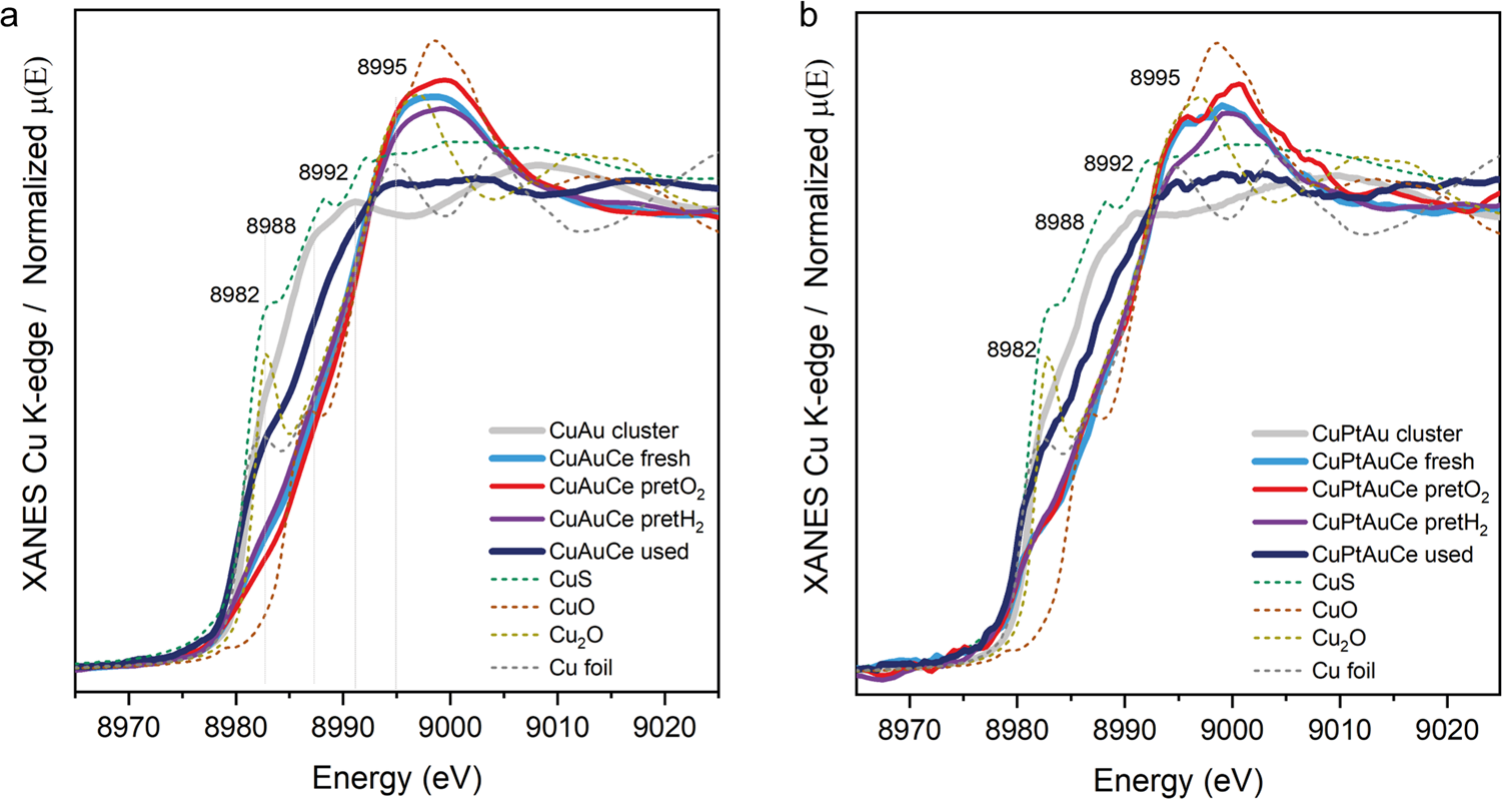
XANES of the Cu K-edge. XANES spectra at Cu K-edge of the CuAu **a** and CuPtAu **b** nanocluster catalysts before the reaction (without pretreatment) and after WGS reaction (used).

Figure 5 shows the Cu K-edge XANES for both bimetallic and trimetallic catalysts at different stages of the study, which include the pre-treatment and reaction phases. Noteworthy are the significant changes in these spectra, especially when comparing the state of the cluster before and after supporting, revealing a strong interaction between the Cu atoms and the CeO_2_ surface. This interaction is to be expected given the placement of the doped Cu atoms at the staple positions in the cluster. One of the most pronounced changes occurs in the pre-edge at 8982 eV, together with the notable intensity variation in the range from 8988 to 9000 eV, indicating a shift towards an oxidized Cu state.

Given the strategic placement of Cu atoms in the stacking structure and our previous observations of ligand migration (as previously reported)^42–44^ to the surface of active oxide supports such as CeO_2_, it becomes plausible that the stacking structure containing Cu, Au and S atoms migrated to the surface of CeO_2_ during different phases, including pretreatments and reaction conditions. Furthermore, the XANES spectra of the catalysts show a more metallic state of Cu after the reaction compared to their previous states. This is an additional indication of the evolving dynamics during the reaction.

**Fig. 6:**
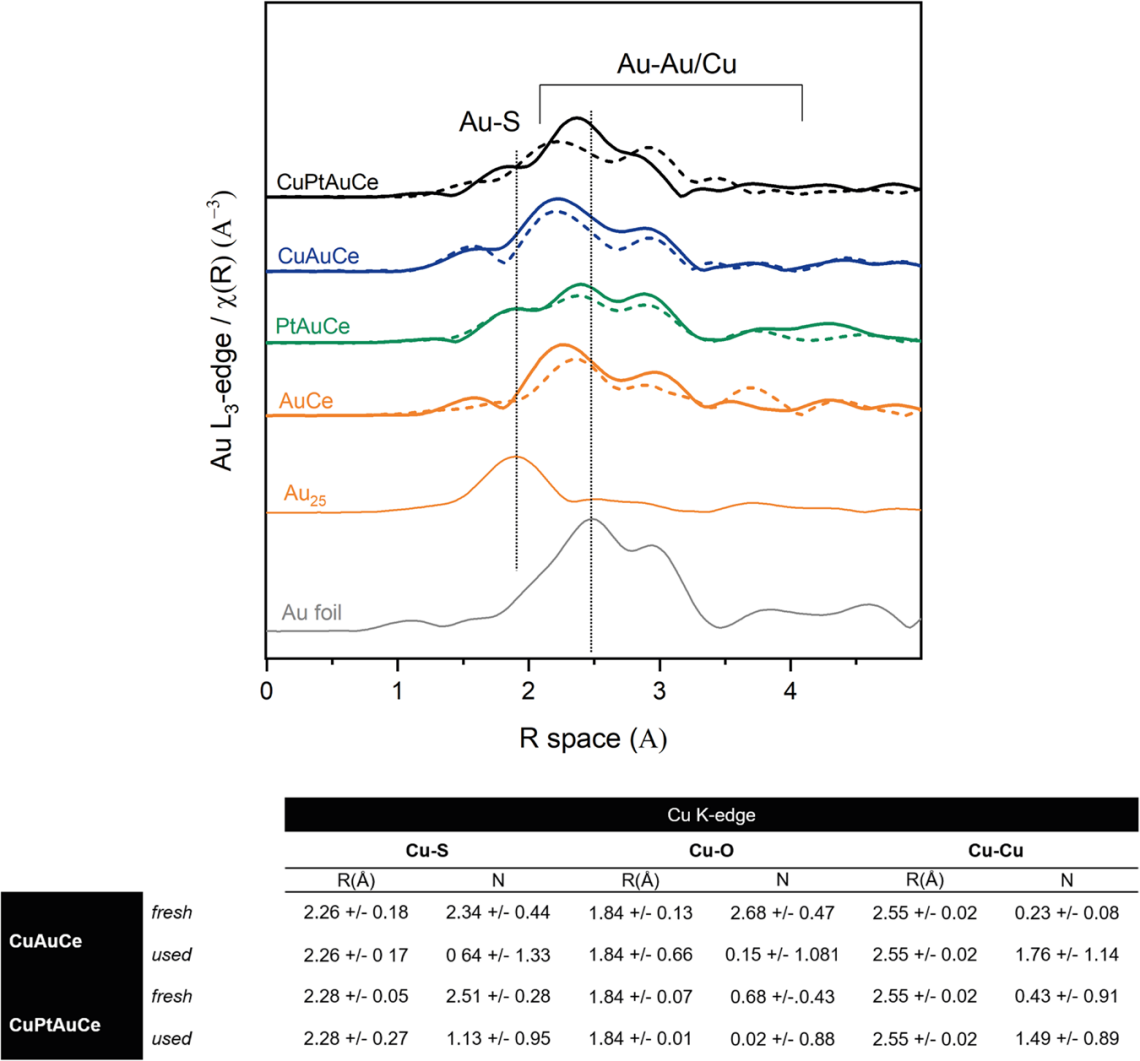
EXAFS of the Cu K-edge. Cu K-edge EXAFS results, figure with R-space of the bimetallic and trimetallic cluster catalysts fresh and after WGS reaction together with the references measured for comparison; table with the EXAFS fitting results (details of fitting in Supporting Information).

The Cu K-edge EXAFS spectra for both CeO_2_-supported samples (as shown in Fig. 6) exhibit features consistent with partially oxidized metallic Cu clusters. Notably, in the fresh samples, an oxidized state of Cu is evident, while a shift towards reduced states during the reaction becoming apparent in the used samples. Additionally, there is an increase in the Cu-Cu CN following the reaction, potentially indicating the formation of small Cu clusters alongside the Au clusters. Consequently, this observation could offer an explanation for why the behaviour of the Cu-doped AuCe catalysts resembles that of the monometallic catalyst in terms of catalytic activity during the WGS reaction.

To corroborate the results obtained from the XAFS studies, we performed infrared measurements with CO as the probed molecule, with the samples before and after the reaction. The CO adsorption bands typically fall within the spectral range of 2200 to 1900 cm^−1^ ^45–47^. In the situation of having three different metals as Au, Cu and Pt, the bands related to the absorption of CO can potentially overlap in this region, therefore in order to identify each of them, a careful analysis of the reference was carried out and summarized in Fig. S4.

**Fig. 7:**
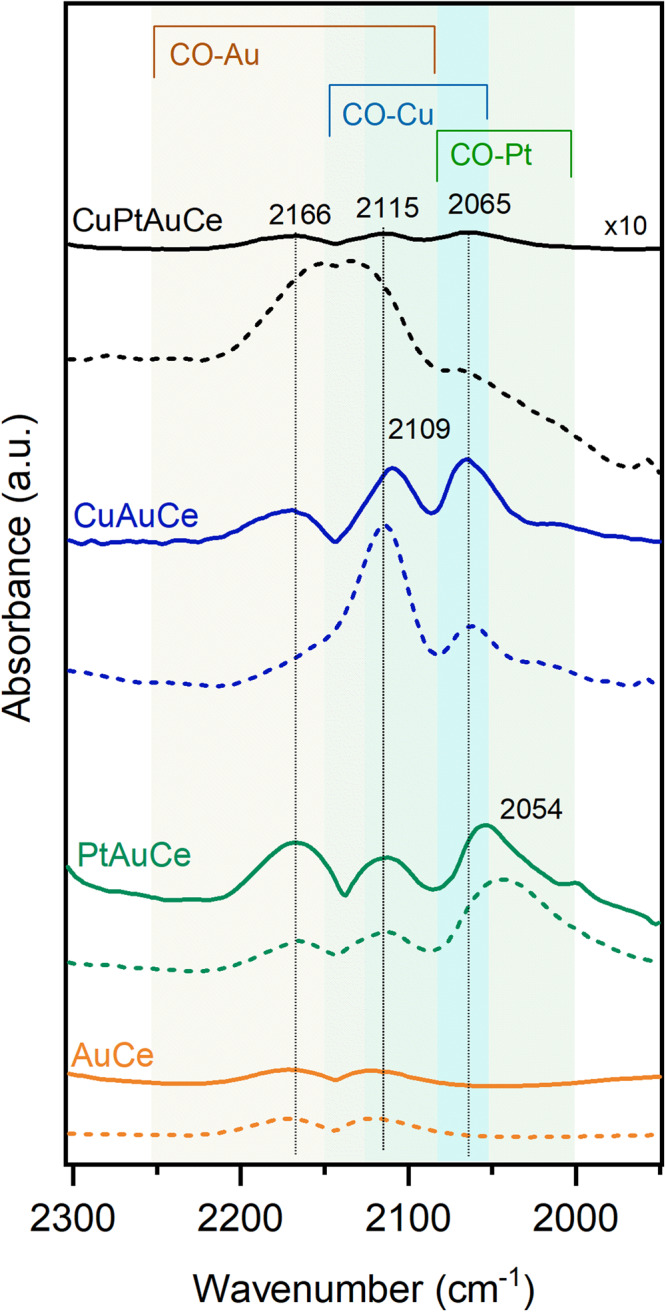
IR spectra of the CO bonding. IR spectra after the CO dosing (CO adsorption experiment) and completed flushing with inert gas once after the pretreatment (dotted line) and once after reaction (solid line) for each catalyst. CO, which stayed on the catalyst surface after each dosing experiment, can be seen. The complete evolution of the CO dosing experiments on the different catalysts can be found in the SI Fig. S4.

Figure 7 shows the different CO vibrations depending on the metals present in each catalyst and their evolution after the reaction. The undoped cluster catalysts, AuCe, present two bands around 2166 and 2115 cm^−1^, related to CO adsorbed on Au^δ+^, which are expected to shift towards higher wavenumbers as the oxidation state of gold increases. The band at 2123 cm^−1^ could then be attributed to CO co-adsorption with oxygen (CO-Au-O)^45^. The interaction between the gold particle and the fully oxidized support leads to the formation of Au-O sites, and the band at 2123 cm^−1^ is typically detected on gold catalysts supported on reduced ceria or titania. On gold nanoparticles supported on different oxides, the component attributed to CO-Au^0^ at 2105 cm^−1^^45^, typical of CO adsorbed on metallic gold, was not detected. However, no change is observed between the sample before and after the reaction, suggesting that the Au nanocluster does not change under the reaction conditions. Similar behaviour is also observed for the PtAuCe catalyst. In agreement with previously reported works^33^, the migration of the Pt atom to the surface of the cluster core after pretreatment is detected by the presence of linearly adsorbed CO between 2090 and 2000 cm^−1^. The PtAuCe catalyst shows three bands with maxima at 2054 cm^−1^, 2112 cm^−1^, and 2166 cm^−1^. The broad bands at 2054 cm^−1^ is particularly interesting as CO adsorption on ceria supported Pt clusters can occur in this range^46^. In addition, positively charged Pt atoms can adsorb CO in higher wave number ranges: 2040–2070 cm^−1^ for Pt^2+^ and 2090–2125 cm^−1^ for Pt^4+^. These regions overlap with the Pt-CO and Au-CO peaks. Based on the spectral differences observed between AuCe and PtAuCe, it is likely that an increased amount of CO-Pt species is present on the PtAuCe catalyst, supporting the proposed migration of Pt to the cluster core surface, which probably contributes to the increased catalytic activity. In addition, previously adsorbed CO appears to be available for reaction on the PtAuCe catalyst.

**Fig. 8:**
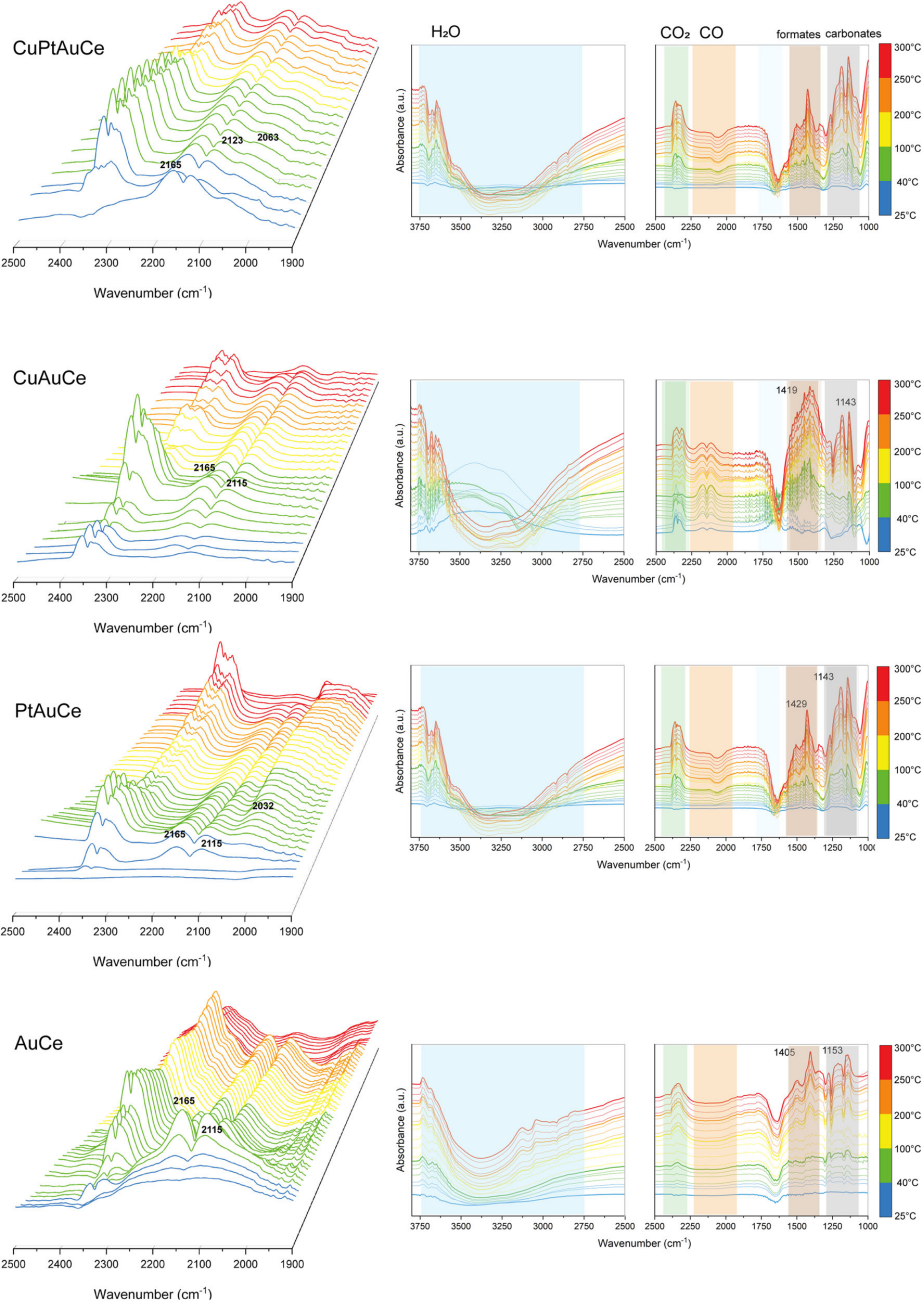
Operando DRIFTS experiments. Operando DRIFTS experiments of the supported nanoclusters, with spectra recorded every 3 min. The system was flushed with Ar ($12{mL}\cdot {\min }^{-1}$) and pretreated as for the catalytic activity tests.

In contrast, the CuAuCe catalyst shows clear spectral changes before and after the reaction (Fig. 8). The CuAuCe spectrum shows three notable maxima at 2168 cm^−1^, 2109 cm^−1^ and 2065 cm^−1^, although the assignment of these peaks can be complicated due to the potential overlap of different species. In addition to CO-Au^0^ complexes, the peak at 2109 cm^−1^ could be attributed to CO-Cu^I^ and/or Au-Cu^+^ complexes. However, considering the spectral context, it seems more plausible that this peak belongs to CO-Au^0^ interactions. In addition, the peak at the lower wavenumber of 2065 cm^−1^ is likely due to interactions between CO and Cu^0^. Conversely, the peak at the higher wavenumber of 2168 cm^−1^ suggests the presence of CO-Au species. The persistence of residual CO on the CuAuCe surface both before and after CO dosing implies that the peak at 2109 cm^-1^ should indeed be attributed to a Cu species such as CO-Cu^I^ and/or Au-Cu^+^. This presence of Cu species at 2109 cm^−1^ and 2065 cm^−1^ could explain the observed increase in catalytic activity, as some CO remains adsorbed on the surface and is available to participate in the reaction.

Significant transformations are particularly evident when the trimetallic CuPtAuCe catalyst is considered during the reaction. The contrast between the spectra acquired before and after CO dosing suggests dynamic changes on the catalyst surface as the reaction unfolds. Within the trimetallic CuPtAuCe catalyst, three distinct peaks appear, suggesting interactions of CO with Au and Cu. Interestingly, these interactions are similar to those observed in the bimetallic CuAuCe catalysts, with no discernible evidence of CO on Pt. This phenomenon could be related to either the coverage of the Cu atoms or their limited migration to the surface due to the trimetallic composition.

### Operando DRIFTS studies during WGS reaction

In order to clarify the variations in catalytic activity and to establish their correlation with potential structural dynamics within the clusters, operando diffuse reflectance infrared Fourier transform spectroscopy (DRIFTS) studies were performed during the WGS reaction. The catalytic activity is strongly related to the evolution of the bands in the region of CO (1900–2200 cm^−1^) and CO_2_ (2400 and 2300 cm^−1^). In the case of the monometallic catalyst *AuCe*, the catalytic activity becomes noticeable only at temperatures above 150°C. This is characterized by the binding of CO to Au and the simultaneous increase of the CO_2_ bands. In order to achieve representative catalytic activity for hydrogen and a reduction in CO_2_ production, temperatures above 250 °C are required, which is consistent with the results of the kinetic tests.

On the other hand, for the PtAuCe catalyst, significant catalytic activity is observed at around 100 °C. This activity increases as the temperature rises, in parallel with the gradual increase in the Pt-CO peak, indicating a continuous supply of CO to the reaction. This robust catalytic performance, supported by the results of kinetic tests, is probably due to the migration of Pt atoms from the core of the cluster to its surface, forming single atomic active sites, in line with our initial hypothesis.

Notably, the CuAuCe catalyst showed a distinct behaviour where the Cu-CO peaks increased up to 200 °C and then decreased. This pattern indicates the involvement of bound CO in the reaction. However, the lack of a significant increase in the CO_2_ binding peak suggests that the reaction is hydrogen oriented, which is consistent with the kinetic results. In contrast, in the case of the trimetallic CuPtAuCe catalyst, Au–CO interactions appear to play a greater role than Cu-CO interactions. Interestingly, there is no detectable appearance of Pt-CO, but a band around 2063 cm^−1^, possibly related to CO on Cu^0^. Therefore, the operando DRIFTS confirmed the hypothesis on the doping atoms evolution observed by the XAFS analysis.

## Conclusions

**Fig. 9:**
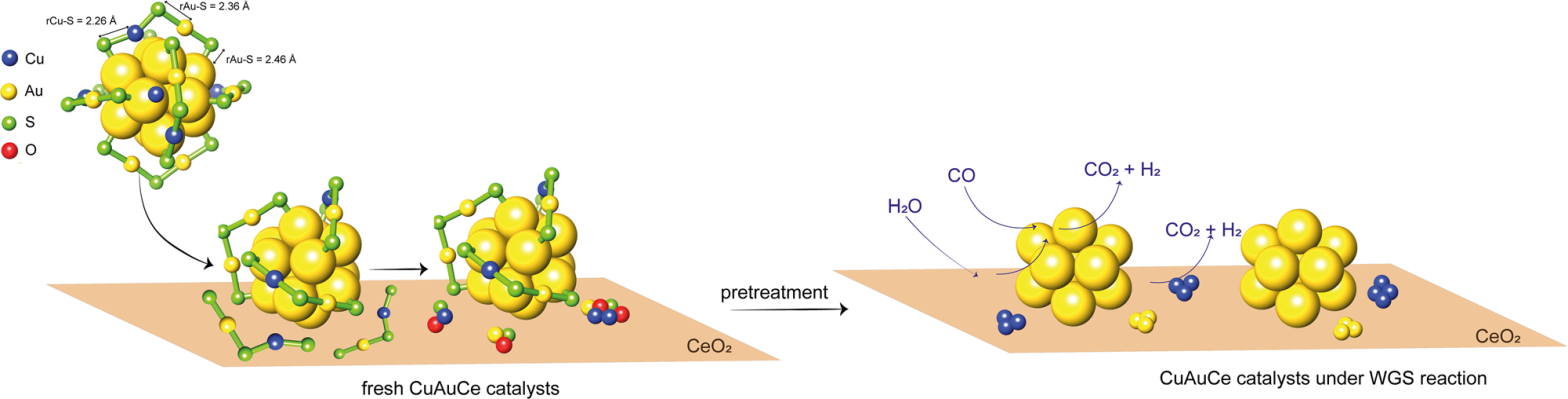
Cu evolution upon supporting of the nanoclusters. Representation of evolution of Cu atoms from the cluster, to the supporting step on CeO_2_ until the reaction conditions. Structure distances are from ref. ^28^.

In conclusion, this study has assessed the complex behaviour of Pt and Cu dopant atoms in Au_25_ clusters supported on CeO_2_ during different phases, including support effects, pretreatment steps and the WGS reaction. Operando XAFS and DRIFTS showed that Cu atoms migrate towards the support and form isolated Cu clusters. In this context, the catalytic activity during the WGS reaction is mainly driven by the stable Au cluster (Fig. 9). Conversely, Pt atoms migrate from the cluster core to the surface, where they stabilize as single atom sites, increasing the catalytic activity and selectivity. When both Pt and Cu are doped into the Au_25_ clusters, their strong interaction with the support in combination with the pretreatment and reaction conditions leads to the growth of the Au clusters. This phenomenon has a detrimental effect on the catalytic behaviour and underlines that in certain cases an excess of metal doping does not necessarily lead to improved catalytic performance.

Understanding the atomic dynamics at different stages of catalyst development is paramount to fine-tuning catalysts and achieving optimal performance. It is crucial to consider the unique properties and location of the individual metal dopants and their interactions with the support and reaction conditions. In summary, this work highlights the intricate interplay of support, metal doping and reaction conditions on the behaviour of Pt and Cu atoms in Au_25_ clusters and provides valuable insights for the rational development of catalysts tailored to specific applications. These findings contribute to the advancement of catalyst science and ultimately facilitate the development of more efficient and selective catalytic systems.

## Methods

The different clusters were prepared based on previous experience and following other modified synthesis protocols described in the supporting information, with the addition of the characterization by ultraviolet-visible spectroscopy (UV-VIS) and matrix assisted laser desorption ionization mass spectrometry (MALDI-MS). To prepare the catalysts, the different Au nanoclusters were supported on the CeO_2_ by dissolving both in toluene and stirring the solution overnight with a wet impregnation. The finished catalysts were characterized using total reflection X-ray fluorescence (TXRF). The catalysts were pretreated by two consecutive oxidations (under O_2_)/reduction (under H_2_) treatments before the WGS reaction kinetic tests were performed at different temperatures (detailed description in supporting information). The structural evolution of the supported clusters was investigated by operando XAFS studies at Au L_3_-edge and Cu K-edge, and complementary characterization on the heteroatom metal dynamics during pretreatment and reaction was obtained by operando DRIFTS. Moreover, theoretical XAFS simulations were conducted. The details of these studies, as well as complementary results, are described in the supporting information, under Supplementary Methods.

### Supplementary information

Supplementary Information

## Data Availability

The data that support the findings of this study are available from the corresponding author upon reasonable request.
