# Supplementary material for: Dynamic behaviour of platinum and copper dopants in gold nanoclusters supported on ceria catalysts
Source: Commun Chem. 2023 Dec 18;6:277. doi: 10.1038/s42004-023-01068-0 (PMC10728199; doi:10.1038/s42004-023-01068-0)
Supplement: Supplementary file 1 — Supplementary Information [file 42004_2023_1068_MOESM1_ESM.pdf]

**Supporting Information**

# Dynamic Behaviour of Platinum and Copper Dopants in Gold Nanoclusters Supported on Ceria Catalysts

Nicole Müller[1#], Rareş Banu[1#], Adea Loxha[1], Florian Schrenk[1,2], Lorenz Lindenthal[1,2], Christoph Rameshan[1,2], Ernst Pitternauer[3], Jordi Llorca[4], Janis Timoshenko[5], Carlo Marini[6], Noelia Barrabés[1*]

*[1]Institute of Materials Chemistry, TU Wien, Getreidemarkt 9/165, 1060 Vienna, Austria*
*[2.] Chair of Physical Chemistry, Montanuniversität Leoben, Franz-Josef-Straße 18, 8700 Leoben, Austria*
*[3]Institute of Analytics, TU Wien, Getreidemarkt 9/165, 1060 Vienna, Austria*
*[4]Institute of Energy Technologies and Department of Chemical Engineering, Universitat Politècnica de Catalunya, EEBE, Eduard Maristany 10-14, 08019 Barcelona, Spain*
*[5] Department of Interface Science, Fritz-Haber Institute of the Max Planck Society, 14195 Berlin, Germany*
*[6.] ALBA Synchrotron Light Facility, Carrer de la Llum 2-26, 08290 Cerdanyola del Valles, Barcelona, Spain*

corresponding author email: noelia.rabanal@tuwien.ac.at

## Supplementary Methods

**Synthesis of $Au_{25}(SC_2H_4Ph)_{18}$.:** The synthesis was carried out following a protocol by Truttmann *et al*. [1] 50ml of THF and 1,270 mmol of $HAuCl_4 \cdot 3H_2O$ were mixed with 1,524 mmol of TOAB and stirred for 10 min. Then, 0,85 mL of 2-phenyl-ethanethiol was added to the solution and stirred until transparent. 12,97 mmol of $NaBH_4$ in 10 ml of ice-cold water was added at once, leading to a dark brown reaction mixture. The solution was stirred for 4 days under ambient conditions, before the solvent was evaporated and the precipitate was washed several times with methanol. The clusters were then separated by Size Exclusion Chromatography (SEC) and their purity evaluated by Ultraviolet-Visible (UV-Vis) spectroscopy and matrix-assisted laser desorption ionization (MALDI) mass spectrometry.

***Synthesis of bi- and trimetallic $M_xAu_{25-x}(SC_2H_4Ph)_{18}$ (M=Pt,Cu)***

**Synthesis of $Cu_xAu_{25-x}(SC_2H_4Ph)_{18}$:** The cluster was prepared according to a procedure presented by the Negishi group. [2] 0,484 mmol $HAuCl_4$•$3H_2O$ were mixed with 0,123 mmol $CuCl_2$•$2H_2O$ and 0,661 mmol TOAB were dissolved in 50 mL of MeOH. The solution was stirred at room temperature for 15 min. Subsequently, 0,963 mL 2-phenyl-ethanethiol (2-PET) were added to the mixture, which was then stirred for further 15 min at room temperature. The reaction mixture was then reduced with 6 mmol $NaBH_4$ dissolved in 10 mL of ice-cold water, yielding a black precipitate, which was stirred for 3 hours at room temperature. Subsequently, the solvent was removed by evaporation and the precipitate washed 4 times with methanol. The product was extracted with acetonitrile and further purified by size exclusion chromatography and analyzed by UV-Vis and MALDI-MS.

**Synthesis of $PtAu_{24}(SC_2H_4Ph)_{18}$:** The PtAu cluster was prepared according to a procedure published by Annelise *et al.* [3] For this, 0,425 mmol $H_2PtCl_6$•$6H_2O$ and 0,017 mmol TOAB were mixed with 0,364 mmol of $HAuCl_4$•$3H_2O$ solution and 60 mL of THF and stirred for 30 min. Subsequently, 0,690 mL 2-PET were added, and the solution was stirred for another 10 min. Next, the mixture was reduced with 20,59 mmol $NaBH_4$ dissolved in 10 mL ice cold water. The solution was stirred for further 4 hours, after which the solvent was removed under reduced pressure and the precipitate was washed 3 times with MeOH and then with acetonitrile and acetone. The crude product is then dissolved in DCM and dried under reduced pressure, after which it was redissolved in THF and filtered using a syringe filter. The product was then dried under reduced pressure and purified by size exclusion chromatography. The final product was characterized by UV-Vis and MALDI-MS.

**Synthesis of $Cu_xPtAu_{24-x}(SC_2H_4Ph)_{18}$:** For the generation of the trimetallic cluster $Cu_xPtAu_{24-x}(2\text{-PET})_{18}$ a specially made Cu ligand was joined with the previously synthesized Pt-cluster, according to previous works.[3] To make the Cu-ligand, two solutions (Solution A and Solution B) were combined. For Solution A 0,356 mmol of $CuCl_2$•$2H_2O$ was combined with 1 mL of water and 5 mL of MeOH in a vial. The mixture is stirred until the color turned bright green. In a separate vial, 0,600 mL of 2-PET, 7 mL of EtOH, and 2 mL of ethylenediamine were mixed. The two solutions are then homogenized and stirred until a light turquoise solid is produced. 0.0002 mmol of the Pt-cluster is then dissolved in 5 mL of toluene. The deep green liquid is then mixed with 0.0041 g of the previously produced Cu-ligand, resulting in a cloudy solution. After stirring for 20 minutes, the reducing agent $NaBH_4$ is added, and the liquid is stirred again. The solvent is then removed under reduced pressure. Size exclusion chromatography was used to purify the nanoclusters, which were then characterized by UV-Vis and MALDI-MS.

## Cluster Characterization (MALDI-MS and UV-Vis)

**UV-Vis spectra** of nanoclusters dissolved in $CH_2Cl_2$ were recorded on a Perkin Elmer Lambda 750 UV-Vis spectrometer.

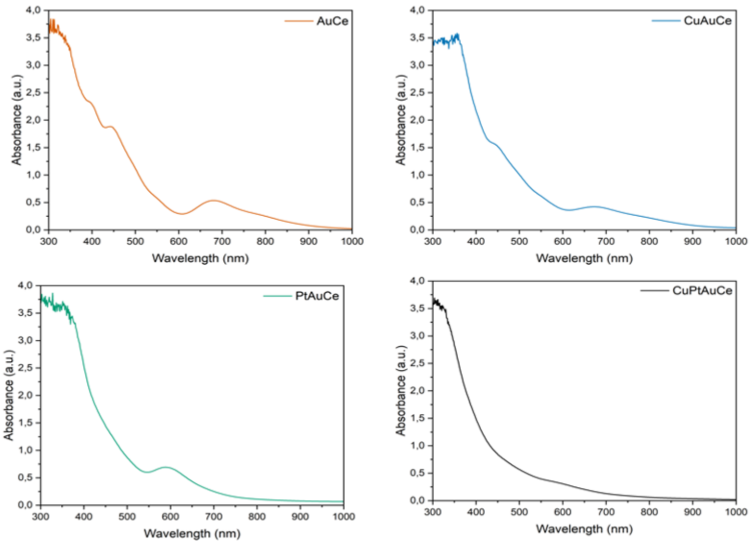


Figure S1. UV-Vis spectra of prepared nanoclusters

All matrix-assisted laser desorption ionization mass spectrometry **(MALDI-MS)** mass spectrometric measurements were performed using a reflectron (RTOF) mass spectrometer (Shimadzu). For analytical experiments, 2,4,6-trihydroxyacetophenone (Sigma-Aldrich) was selected as MALDI-MS matrix. MALDI-RTOF mass spectra were acquired near threshold laser irradiance to obtain mass spectra of sufficient mass spectrometric resolution [3000–5000 at full width half-maximum (fwhm)]. All displayed mass spectra were based on averaging 300–600 single and unselected laser pulses (λ = 337 nm at 50 Hz).

Note: high fragmentation of the cluster lead to not clear MALDI-MS signal (no MALDI-MS of the CuAuCe cluster could be measured)

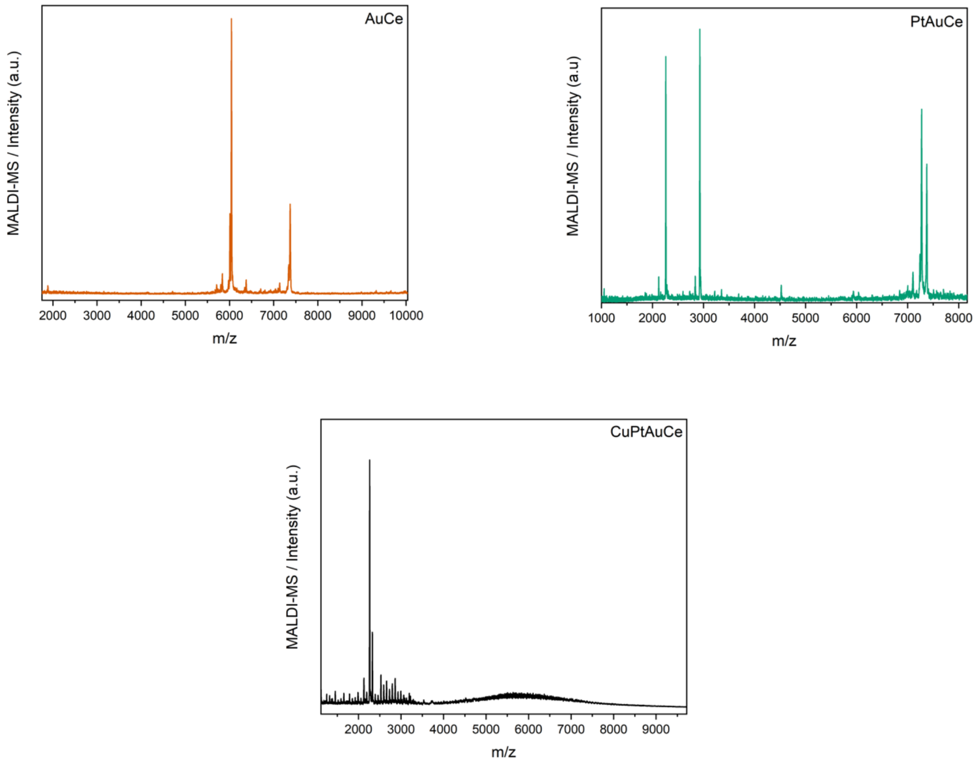


Figure S2. MALDI results of the prepared clusters

## Catalyst preparation

The deposition of the prepared nanoclusters on the ceria was performed according to previous experience. [1-4] The nanoclusters were dissolved in toluene and mixed with a suspension of ceria in toluene for 24 hours. Next, the mixtures were centrifuged and the dried. The success of the deposition was controlled *via* TXRF.

## TXRF results

Using total reflection X-ray fluorescence spectroscopy (TXRF) in combination with an ATOMIKA 8030C X-ray fluorescence analyzer, the exact metal loading (wt%) of the gold nanocluster catalysts was measured. Using 1 mg of sample combined with 5 l of a 1% polyvinyl alcohol solution, samples were affixed to total reflecting quartz reflectors (for fixation). Prior to measuring each specimen, blank reflector measurements were performed to prevent cross-contamination. The measured elements (Au and Ce) have detection limits between 10 and 100 µg/g.

Table S1. XRF results of the supported nanoclusters catalysts

| Amount of dopant atom related to $CeO_2$ amount in $wt\%$ | | | | |
|---|---|---|---|---|
| Catalyst | Au | Pt | Cu | Total |
| $Au_{25}$(2-PET)/$CeO_2$ | 0.58 | - | - | 0.58 |
| $Cu_xAu_{25-x}$(2-PET)/$CeO_2$ | 0.26 | - | 0.06 | 0.32 |
| $PtAu_{24}$(2-PET)/$CeO_2$ | 0.36 | 0.11 | - | 0.47 |
| $Cu_xPtAu_{24-x}$(2-PET)/$CeO_2$ | 0.14 | 0.04 | 0.05 | 0.23 |

## Transmission electron microscopy

High-resolution transmission electron microscopy (HRTEM) and high-angle annular dark-field scanning transmission electron microscopy (HAADF-STEM) images were obtained by using a field emission gun FEI Tecnai F20 microscope operated at 200 kV with a point-to-point resolution of 0.19 nm.

## Catalytic activity studies

A flow reactor connected to a micro-gas chromatograph (Micro-GC, Fusion 3000 A, Inficon) was used to perform the kinetic tests of the Au nanocluster catalysts during WGSR. The temperature was measured and controlled by using a Ni/NiCr thermocouple that was linked to a PID controller of a cylindrical oven. The catalyst powder, 25 mg of each catalyst, was transferred into a quartz glass reaction tube and the ends were closed with glass wool. The reaction tube was then placed into the cylindrical oven and then flushed with a flow of 20 ml/min of Ar gas. For all catalysts an oxidative and then a reductive pretreatment was performed with a 10 °C/min temperature ramp till 300 °C. The Pret$O_2$ (5 % $O_2$ in Ar, 20 ml/min total gas flow) is used to remove pollutants and residue from the surface of the catalyst, as well as ligands that are left over after syntheses. The Pret$H_2$ (5 % $H_2$ in Ar, 20 ml/min total gas flow) initiates the reduction of noble metals, such as Pt and Au, which are in their oxidized state. [4] At the maximum temperature of 300 °C the catalysts were soaked for 30 minutes in the respective pretreatment gas mixture before they were cooled down to room temperature and flushed with a flow of 20 ml/min of Ar gas again. For the reaction the sample was soaked with the reaction mixture (5% CO and 1.84% of $H_2O$ in Ar, 21 ml/min total gas flow) for 15 min at room temperature for equilibration, then heated up with a ramp rate of 5 °C/min and then soaked for

10 minutes each at 100 °C, 200 °C and 250 °C. At the maximum temperature of 300 °C the sample was soaked with the reaction mixture for 30 minutes before it was cooled down again to room temperature with a flow of 20 ml/min of Ar gas. To compare the activity of the Au nanocluster catalysts, the results were normalized to 25 mg catalyst with a cluster loading of 0.5 wt%. The pure $CeO_2$ support was also examined to learn how much itself contributes to the activity of the catalysts. [4, 5]

## Catalytic activity in the WGS reaction

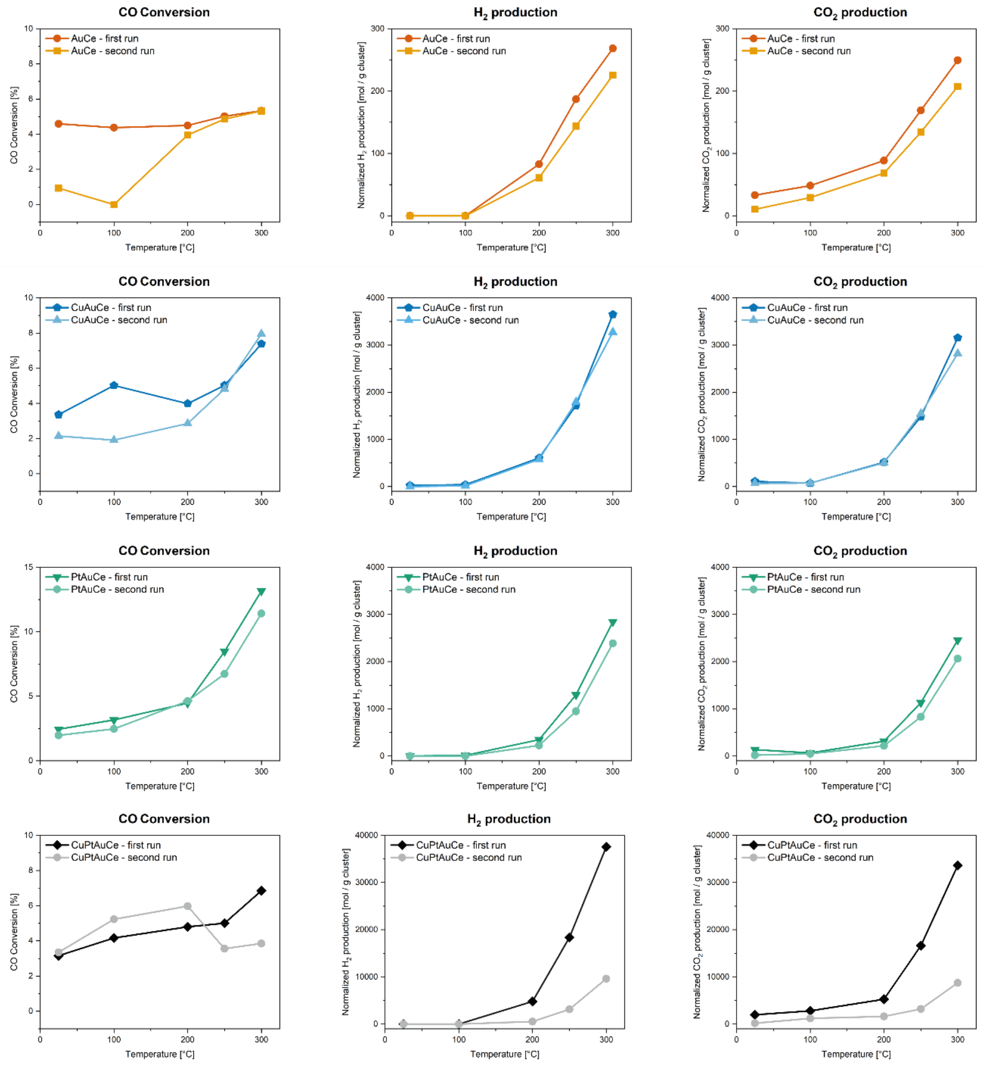


Figure S3. Stability studies with the nanocluster catalysts in the WGS reaction

## Spectroscopic studies

### In situ IR DRIFTS

Operando infrared studies (transmission FTIR) were conducted using a Bruker Vertex 70 spectrometer. The catalyst, approximately 10 mg, was transferred into the flow cell (PIKE Technologies DiffusIR). The gas flow was controlled by a mks Multi gas controller 647C and the temperature was controlled with X and a Liquid Recirculator (PIKE Technologies). The IR spectra were recorded during all the measurements in a 3-minute interval. The system was flushed with a flow of 12 ml/min of Ar and then pretreated as described in the catalytic activity

studies with Pret$O_2$ (5 % $O_2$ in Ar, 13 ml/min total gas flow) and with Pret$H_2$ (5 % $H_2$ in Ar, 12 ml/min total gas flow). After the pretreatment took place a CO adsorption experiment was performed. The sample was exposed to 2 ml/min of CO in Ar (12 ml/min total flow rate) until the IR band of CO stopped changing substantially. Next, 12 ml/min of Ar was circulated through the cell until there were no more changes in the IR spectra. After completing the CO adsorption experiment the catalyst was measured while undergoing the WGSR a gas mixture containing 5.55% CO (due to a limited gas flow controller) and 1.84% of $H_2O$ in Ar (18 ml/min total gas flow) and the same heating scheme that is described in the catalytic activity studies. After the reaction finished and the system was then flushed again with Ar and cooled to room temperature the CO adsorption experiment was repeated.

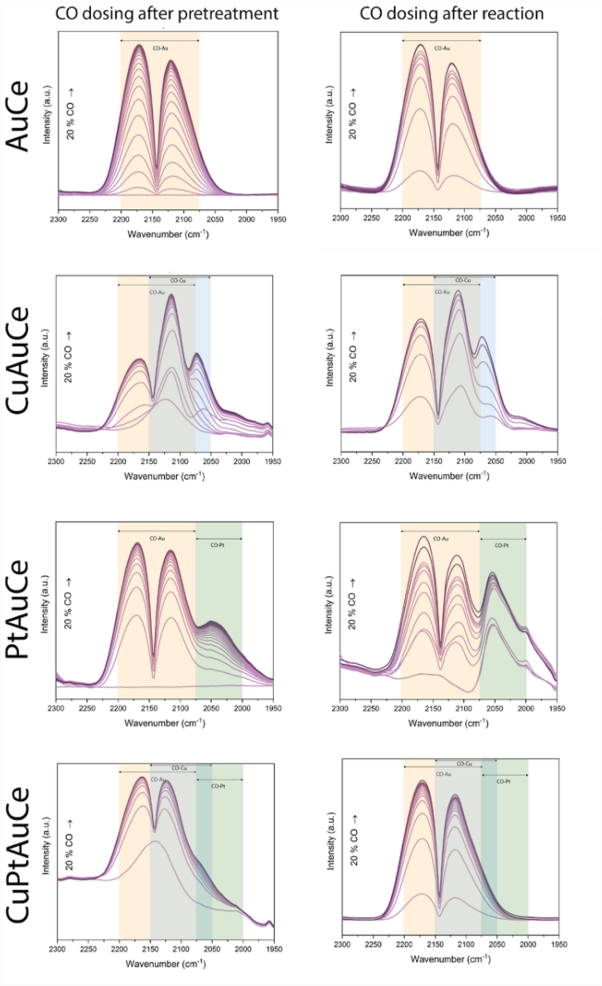


| Compound | Wavenumber ($cm^{-1}$) |
|---|---|
| **CO-Au (general area)** | **2200 - 2075** |
| CO-Au-O | 2120 - 2080 |
| CO-$Au^0$ | 2105 |
| CO-$Au^{\delta+}$ | 2130 - 2175 |
| **CO-Cu (general area)** | **2150 – 2050** |
| CO-$Cu^0$ | < 2100 |
| CO-$Cu^I$ | 2120 |
| Au-$Cu^+$ | 2125 |
| **CO-Pt (general area)** | **2125 – 2000** |
| CO-$Pt^0$ | 2045 |
| CO-$Pt^{\delta+}$ | > 2045 |

Figure S4. CO dosing IR experiments with the nanoclusters catalysts and table of infrared band assignments

### ***In-situ / operando*** **XAFS**

X-ray Absorption Spectroscopy (XAS) measurements were performed at the CLAESS Beamline at Alba Synchrotron in fluorescence mode (Cu K-edge and Au-L3 edge) in the beamline's solid/gas reactor multipurpose cell. The catalysts were pressed into pellets. The samples were pretreated inside the multipurpose cell at 250 °C for 40 min under oxygen flow (pretO2; 5% O2 in He, total flow: 45 ml/min; cool down 40 ml/min He) followed by a reductive pretreatment under hydrogen (pretH2; 5% H2 in He, total flow: 45 ml/min) at the same conditions. After cooling down after the pretH2, the gas mixture was changed to reaction conditions (reaction: 5% CO, 1.84% H2O in He, total flow: 45 ml/min). The samples were

heated to 300 °C with a ramp of 5 °C/min. The maximum temperature was held for 30 min, and then the reaction chamber was cooled down to RT (45 ml/min He). Extended X-ray Absorption Fine Structure (EXAFS) spectra were taken at 40 °C in He at the beginning, after pretreatment and after reaction (for every important step) for each sample, without opening the reaction chamber in between.

**Beamtime details:** Spectra have been collected at CLAESS, the XAS and XES dedicated beamline of Spanish light source, ALBA. The synchrotron radiation emitted by a wiggler source was monochromatized using a double crystal Si(311) monochromator. The rejection of higher harmonics was done by choosing proper angles and coatings of the collimating and focusing mirrors. XAS measurements were performed in fluorescence continuous mode using a multi-element silicon drift detector with Xspress3 electronics. Slit gap in front of I0 ionization chamber have been set to different values in order to guarantee the correct deadtime values depending on the sample measured. Energy scale at Au L3 and Cu K edges have been previously calibrated by measuring the Au and Cu foils respectively. To correctly catch the XANES features of the spectra, an energy step of 0.3 eV has been chosen in the XANES region, differently from the EXAFS region sampled at 1eV.

**EXAFS fitting procedure:** XAS data have been processed according to standard procedure. Au $L_3$ edge spectra have been normalized by calculating and subtracting pre-edge and post-edge backgrounds as low order polynomial smooth curves. The corresponding EXAFS signal has been then extracted, k-squared weighted, and Fourier transformed (FT). $Au_{25}$, CuS, and $PtAu_{24}$ and $Cu_5Au_{20}$ clusters have been considered as the starting point for EXAFS data modelling, providing the theoretical phases and amplitudes of the scattering paths by means of self-consistent ab-initio calculations performed with FEFFlite code. [6] To get more robust determination of the EXAFS structural parameters, we fitted all Au $L_3$-edge EXAFS spectra (28 spectra in total), and all Cu K-edge (16 spectra in total) simultaneously. The fits have been performed using a customized code based on IFEFFIT one. [7]

### *Au L3-edge EXAFS fit*

A three shell model (with Au-S, Au-Au, and Au-Cu contributions) has been considered to model the data. For each spectra of the dataset we considered as fitting parameters three coordination numbers CN ($N_{Au\text{-}S}$, $N_{Au\text{-}Au}$, and $N_{Au\text{-}Cu}$) and two correction factors for interatomic distances ($\Delta R_{Au\text{-}S}$ and $\Delta R_{Au\text{-}Au} = \Delta R_{Au\text{-}Cu}$), which means that we constrained Au-Cu and Au-Au distances. Due to the limited k range available and the strong inter-correlation between the fitting parameters, we decided to use only two disorder parameters ($\sigma^2_{Au\text{-}S}$ and $\sigma^2_{Au\text{-}Au} = \sigma^2_{Au\text{-}Cu}$) kept common for all the spectra in the dataset. Such assumption can be justified considering the limited temperature range of these in-situ measurements (25-250 C) and by the fact that in general the static structural disorder is higher than the temperature one for small clusters. [8] This approach assures that the coordination numbers obtained from the analysis are consistent with all the experimental information available. Finally we fit the energy correction to photoelectron reference ΔE0 and the passive electron reduction factors $S0^2$, again common to all the spectra.

According to standard EXAFS analysis practices, the maximum number of independent variables ($N_{th}$) in the EXAFS fit is given by the formula: [9]

$$N_{th} = \frac{2\Delta k \Delta R}{\pi} \times N_{spectra}$$

Where $\Delta k$ and $\Delta R$ represent the k and r ranges respectively and $N_{spectra}$ is the number of spectra in the dataset. In this case we got $\Delta k$ = 7.2 Å$^{-1}$, $\Delta R$ = 2.3 Å, and $N_{spectra}$ = 28 which means:

$$N_{th} = \frac{2(7.2)(2.3)}{\pi} \times 28 = \frac{33.12}{\pi} \times 28{\sim}295$$

While the total number of parameter used N is:
N = (3 CN + 2 ΔR)×28 + $\sigma^2_{Au\text{-}S}$ + $\sigma^2_{Au\text{-}Au}$ + $S0^2$ + ΔE0 = 144.

Table S2. Parameter fitting EXAFS Au $L_3$-edge

| amp | Enot(S) | Enot(Au) | Enot(Cu) | $\sigma^2$(Au–P) | $\sigma^2$(Au–S) | $\sigma^2$(Au–Au) |
|---|---|---|---|---|---|---|
| 0.77±0.22 | 3.39±0.25 | 0.43±0.52 | 8.95±2.25 | 0.013±0.002 | 0.004±0.001 | 0.010±0.000 |

### *Cu K-edge EXAFS fit*

A three shells model (with Cu-S, Cu-O and Cu-Cu contributions,) has been considered to model the EXAFS data at Cu K edge. For each spectra of the dataset, we considered as fitting parameters three coordination numbers CN ($N_{Cu\text{-}S}$, $N_{Cu\text{-}O}$,$N_{Cu\text{-}Cu}$) and three correction factors for interatomic distances ($dR_{Cu\text{-}S}$, $dR_{Cu\text{-}O}$, $dR_{Cu\text{-}Cu}$). Also in this case, we decided to use only three disorder parameters ($\sigma^2_{Cu\text{-}S}$, $\sigma^2_{Cu\text{-}O}$, and $\sigma^2_{Cu\text{-}Cu}$) for the whole dataset (see previous discussion in the Au L3 edge section), plus three energy correction to photoelectron reference ΔE0 (one for each bounding species) and one unique passive electron reduction factors $S0^2$. The maximum number of independent variables ($N_{th}$) in this EXAFS fit is thus:

$$N_{th} = \frac{2\Delta k \Delta R}{\pi} \times N_{spectra}$$

where $\Delta k$ = 10 Å$^{-1}$, $\Delta R$ = 3 Å, and $N_{spectra}$ = 12. This which means:

$$N_{th} = \frac{2(10)(3)}{\pi} \times 12 = \frac{60}{\pi} \times 12{\sim}229$$

While the total number of parameter used N is:
N = (3 CN + 3 δR)12 + $\sigma^2_{Cu\text{-}S}$ + $\sigma^2_{Cu\text{-}O}$ + $\sigma^2_{Cu\text{-}Cu}$ + $S0^2$ + 3 ΔE0 = 79

Table S3. Parameter fitting EXAFS Cu K-edge

| amp | Enot(S) | Enot(O) | Enot(Cu) | $\sigma^2$(Cu-Cu/Au) | $\sigma^2$(Cu–S) | $\sigma^2$(Cu–O) |
|---|---|---|---|---|---|---|
| 0.77±0.44 | 3.33±0.45 | 0.56±0.45 | -8.26±0.42 | 0.004±0.002 | 0.007±0.003 | 0.010±0.003 |

Table S4. EXAFS fitting results

| Au $L_3$-edge | | | | | |
|---|---|---|---|---|---|
| | | Au-S | | Au-Au | |
| | | R (Å ) | N | R (Å ) | N |
| **$Au_{25}$** | *cluster* | 2.31 +/- 0.13 | 1.21 +/- 0.28 | 2.78 +/- 0.09 | 1.75 +/- 0.64 |
| **AuCe** | *fresh* | 2.30 +/- 0.12 | 2.06 +/- 0.16 | 2.86 +/- 0.07 | 2.22 +/- 0.89 |
| | *pret ($O_2$+$H_2$)* | 2.30 +/- 0.12 | 0.2 +/- 0.76 | 2.79 +/- 0.05 | 6.60 +/- 1.33 |
| | *used* | 2.27 +/- 0.14 | 0.15 +/- 0.65 | 2.78 +/- 0.07 | 7.04 +/- 0.78 |
| **PtAuCe** | *fresh* | 2.30 +/-0.10 | 2.20 +/- 0.11 | 2.77 +/- 0.07 | 0.52 +/- 0.61 |
| | *pret$O_2$* | 2.27 +/- 0.11 | 0.37 +/-0.34 | 2.78 +/- 0.01 | 5.80 +/- 0.41 |
| | *Pret$H_2$* | 2.30 +/- 0.11 | 0.38 +/- 0.46 | 2.78 +/- 0.07 | 6.24 +/- 0.65 |
| | *used* | 2.30 +/- 0.11 | 0.42 +/- 0.22 | 2.78 +/- 0.01 | 8.75 +/- 0.36 |
| **$CuxAu_{25-x}$** | *cluster* | 2.31 +/- 0.18 | 2.97 +/- 0.34 | 2.80 +/- 0.07 | 3.06 +/- 0.60 |
| **CuAuCe** | *fresh* | 2.31 +/- 0.20 | 2.48 +/- 0.35 | 2.79 +/- 0.07 | 1.07 +/- 0.67 |
| | *pret$O_2$* | 2.27 +/- 0.10 | 0.74 +/- 0.17 | 2.81 +/- 0.01 | 7.52 +/- 0.56 |
| | *Pret$H_2$* | 2.29 +/- 0.1 | 0.20+/- 0.58 | 2.80 +/- 0,01 | 7.60 +/- 0.31 |
| | *used* | 2.29 +/- 0.1 | 0.09 +/- 0.13 | 2.77 +/- 0.04 | 7.22 +/- 0.62 |
| **$CuxPtAu_{24-x}$** | *cluster* | 2.30 +/-0.13 | 1.87 +/- 0.13 | 2.81 +/-.0.06 | 0.36 +/- 0.83 |
| **CuPtAu24Ce** | *fresh* | 2.30 +/- 0.17 | 2.97+/- 0.34 | 2.75 +/- 0.09 | 3.06 +/- 0.60 |
| | *pret$O_2$* | 2.30 +/- 0.17 | 0.29 +- 0.23 | 2.79 +/- 0 07 | 6.15 +/- 0.55 |
| | *Pret$H_2$* | 2.32 +/- 0.14 | 0.41 +/- 0.33 | 2.79 +/- 0.09 | 7.13 +/- 0 85 |
| | *used* | 2.39 +/- 0.21 | 0.17 +/- 0.66 | 2.76 +/- 0.10 | 10.13 +/- 0.64 |

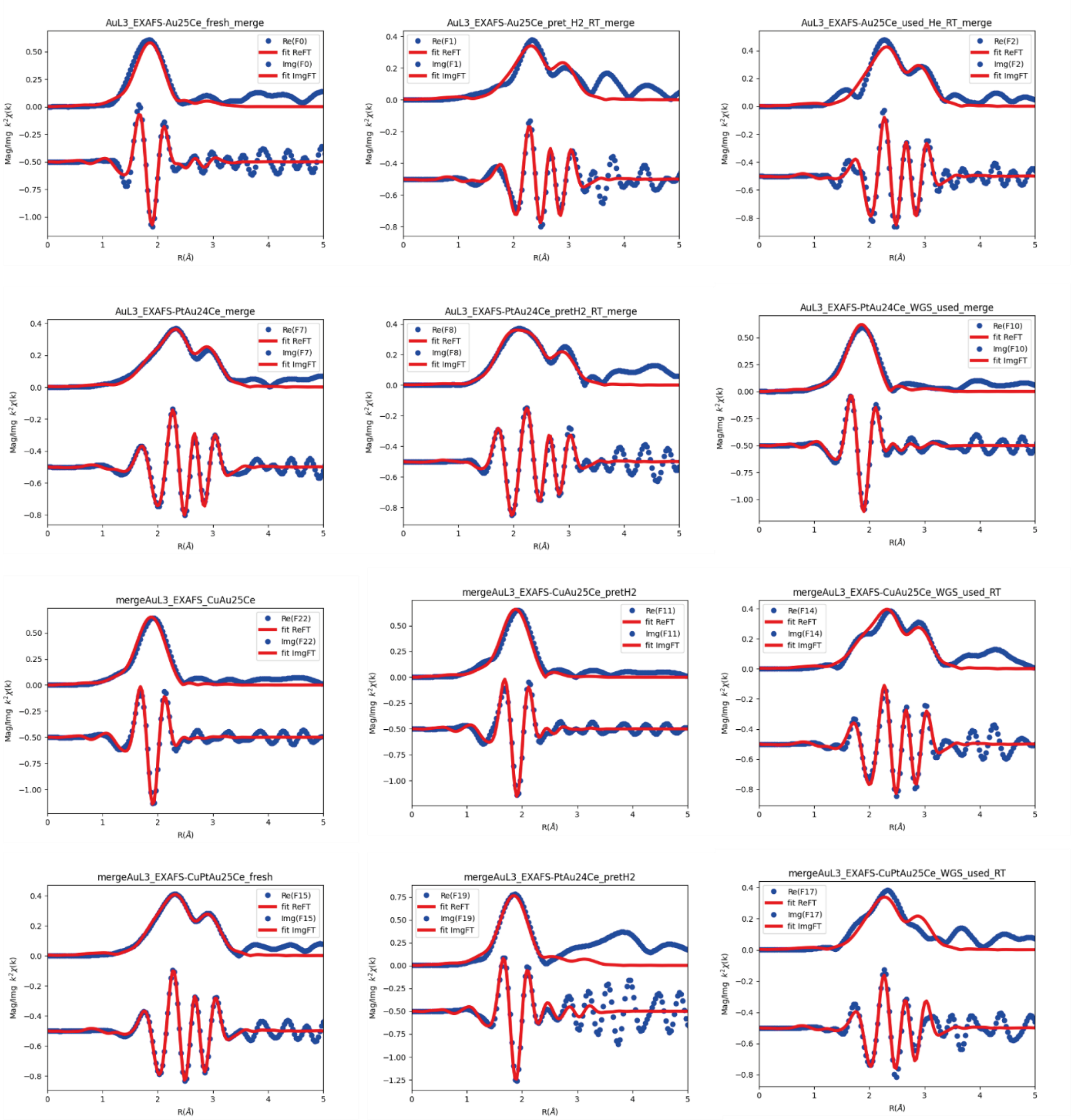


Figure S5. Representative examples of Au $L_3$-edge fitting

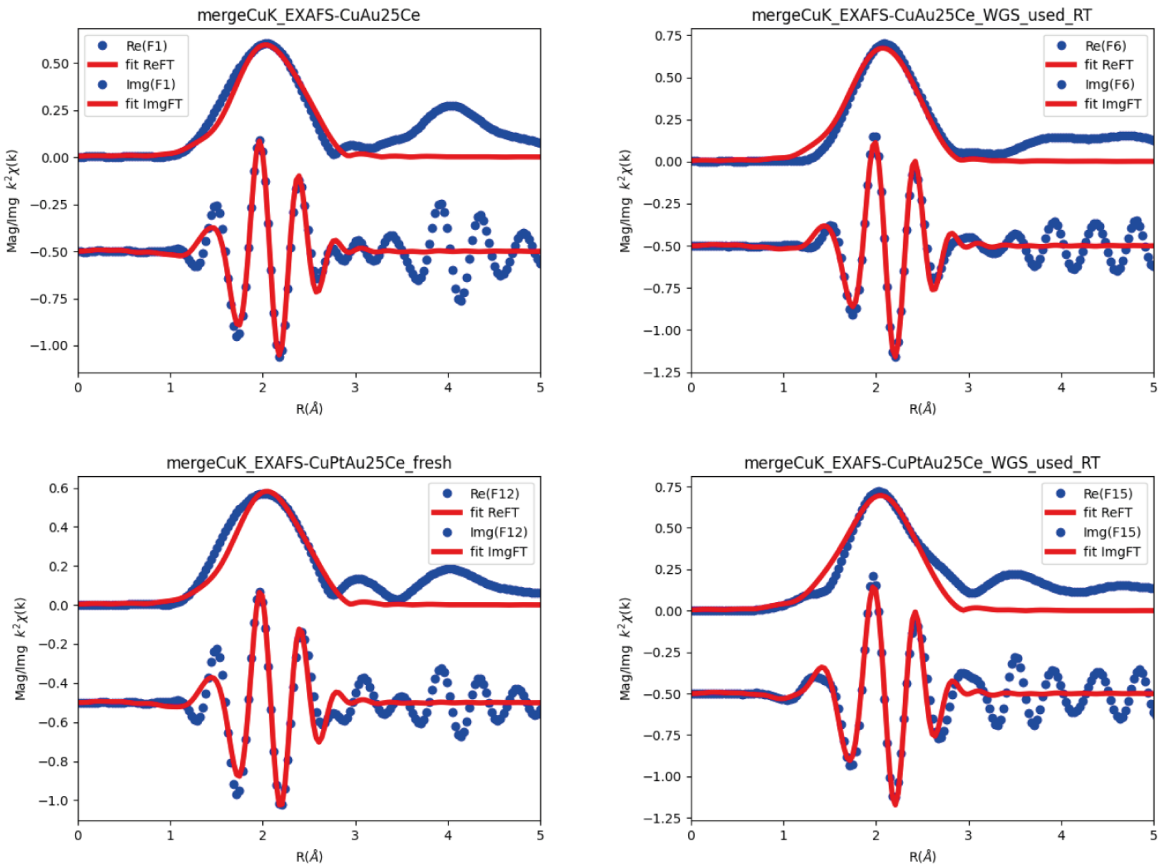


Figure S6. Representative examples of Cu K-edge fitting

## References


1. Truttmann, V., et al., *CeO2 Supported Gold Nanocluster Catalysts for CO Oxidation: Surface Evolution Influenced by the Ligand Shell.* ChemCatChem, 2022. **14**(14): p. e202200322.
2. Hossain, S., et al., *Determining and Controlling Cu-Substitution Sites in Thiolate-Protected Gold-Based 25-Atom Alloy Nanoclusters.* The Journal of Physical Chemistry C, 2020. **124**(40): p. 22304-22313.
3. Sels, A., et al., *Isolation of atomically precise mixed ligand shell PdAu24 clusters.* Nanoscale, 2016. **8**(21): p. 11130-11135.
4. Garcia, C., et al., *Dynamics of Pd Dopant Atoms inside Au Nanoclusters during Catalytic CO Oxidation.* The Journal of Physical Chemistry C, 2020. **124**(43): p. 23626-23636.
5. García, C., et al., *Support effect on the reactivity and stability of Au25(SR)18 and Au144(SR)60 nanoclusters in liquid phase cyclohexane oxidation.* Catalysis Today, 2019. **336**: p. 174-185.
6. J.J. Rehr, et al., *Parameter-free calculations of x-ray spectra with FEFF9*. Phys. Chem. Chem. Phys., **12**, 5503-5513 (2010).
7. M. Newville, *IFEFFIT: interactive EXAFS analysis and FEFF fitting*. J. Synchrotron Rad. **8**, pp 322--324 (2001).
8. Likun Wang et al., *Designing Nanoplatelet Alloy/Nafion Catalytic Interface for Optimization of PEMFCs: Performance, Durability, and CO Resistance.* ACS Catal. 2019, 9, **2**, 1446–1456

9. *X-Ray Absorption and X-Ray Emission Spectroscopy: Theory and Applications*. Editor(s): Jeroen A. Van Bokhoven Carlo Lamberti, Print ISBN:9781118844236. Online ISBN:9781118844243 |DOI:10.1002/9781118844243
